# Family Caregiver Suffering in Caring for Patients with Amyotrophic Lateral Sclerosis in Korea

**DOI:** 10.3390/ijerph18094937

**Published:** 2021-05-06

**Authors:** Juyeon Oh, Jung-A Kim, Min Sun Chu

**Affiliations:** 1 College of Nursing, Dankook University, Cheonan 31116, Korea; ohjy@dankook.ac.kr; 2 School of Nursing, Hanyang University, Seoul 04763, Korea; joyhippo@hanyang.ac.kr; 3 Seoul Women’s College of Nursing, Seoul 03617, Korea

**Keywords:** amyotrophic lateral sclerosis, caregivers, family, frustration, health service needs and demand

## Abstract

The purpose of this study was to describe the meaning of the suffering experience of Korean ALS family caregivers. This is a descriptive phenomenological study that included ten participants using convenience sampling with maximum variation in a tertiary hospital in Seoul, South Korea. Colaizzi’s data analysis method was used to inductively determine themes and formulate meanings. The three main themes derived from analysis were: “frustration with seeing a patient suffering”, “burnout at the cost of a life of dedication”, and “desperate need for help”. Caregivers experience high levels of suffering, which can come in various forms depending on the circumstances of the particular patient and family. Especially, distress from seeing a loved one suffering was another aspect of suffering in Korean ALS caregivers, reflecting strong family ties. At the same time, patients are in desperate need of help and support from their families. Thus, it is essential to provide care to lessen the causes of distress and meet the needs of not only patients, but also caregivers through family-centered care to improve overall quality of life for all involved.

## 1. Introduction

Patients with amyotrophic lateral sclerosis (ALS) need help in everyday tasks, and the role of primary caregiver is usually assumed by family members [1]. Previous studies have extensively noted the high burden and low quality of life (QOL) of ALS family caregivers [2,3]. The distress of ALS family caregivers is known to influence not only their own QOL and mental health, but also the QOL of patients [2,4]. The psychological well-being of caregivers has a great influence on their care [5] and patients’ outcomes [6]. Patient management, such as routine check-ups, has been limited in 2020 and 2021 because of the COVID-19 pandemic, which has made it more important to manage patients at home with thorough professionalism. [7].

According to studies conducted in Korea, ALS family caregivers tend to experience a greater burden than their counterparts in Western countries, and have poorer mental health. Oh et al. reported the caregiver burden of Korean ALS caregivers scored by Zarit Burden Interview was higher than European countries [8] and also have higher ratio of depression than US [5]. Although Korea has advanced medical technology and National Health Insurance covers the entire Korean population, Korea has a weaker social infrastructure for chronic diseases than Western countries, and the vast majority of the burden is borne by patients and their families [9]. Additionally, family ties are highly valued because of the roots in Confucianism in Korea. Previous studies reported high levels of caregiver burden and depression and poor mental well-being [5,8,10] among Korean caregivers of ALS; these studies employed quantitative analysis using structured questionnaires to assess those variables, thus providing only a limited understanding of the problems faced by caregivers and failing to specify the areas where distress was felt. Caregiving for patients with rare diseases is qualitatively different from caring for patients with chronic diseases in general. For example, the caregivers of patients with rare cancers experience unique and substantial burdens that arise from limited therapeutic options, delayed diagnosis, lack of information, as well as psychological distress related to uncertainty about the future [11]. Additionally, caregivers of rare diseases experience loneliness because no one knows the disease, and feel desperate with no choice but just look after the patient [12].

Cassel [13] defined suffering as “a state of severe distress associated with events that threaten the intactness of the person”. The concept of pain is different from such concepts as anxiety, grief, and conflict [14]. Understanding the essence of suffering beyond the caregiver burden and its causes is a prerequisite to providing adequate family-centered care to lessen the level of distress and to improve QOL of the whole family. Therefore, the research question for this study was: “What is the essence of the suffering experiences of Korean family caregivers while providing care for ALS patients?”

## 2. Materials and Methods

This qualitative study used descriptive phenomenological methods. As the goal of descriptive phenomenology is to identify the essence of human experience [15], this method was considered as most suitable to explore the meaning and essence of family caregiver suffering.

### 2.1. Data Collection

Secondary analysis of interviews was conducted in the project regarding the supportive care needs of patients and caregivers in Korea [16]. This study re-analyzed interviews with 10 caregivers meeting the following conditions: (1) providing home care for ALS patient, (2) relative of ALS patient, and (3) aged 19 or over. In the primary data collection, 11 caregivers were included using purposive sampling with maximum variation to include persons of diverse family relations, age, and gender. The participants were selected from among caregivers who accompanied patients to a tertiary hospital for medical services. The caregivers were interviewed during or after patient medical treatment. The interviews, each lasting 40 min to 90 min, were conducted in an area separated from the patient. The open-ended interviews provide participants with an opportunity to describe their experience. The open-ended questions included, “Please tell me about frustrating experiences while taking care of your loved one” and “Please tell me about stressful thoughts and feelings that you’ve had about taking care of your loved one”. Although the questions in each interview contained a number of questions related to the supportive needs of patients and caregivers for the primary study, this study selected and analyzed the answers to the above questions. All interviews were audio recorded with brief notes and transcribed into text. Each interview was terminated at a saturated level when sufficient data were acquired and new information was no longer available. Data requiring confirmation or clarification were re-directed to participants. The interviews were conducted from July to August 2016. To maximize the saturation level of the data, after the primary data analysis for this study (in May 2019), two participants were approached to ask if there was anything new that was not described in the results.

### 2.2. Data Analysis

All researchers conducted data analysis based on the seven stage method described by Colaizzi [17]. The transcription was read repeatedly to determine the meaning and essence of suffering experienced by ALS family caregivers, and statements that vividly described experiences were extracted. The meanings behind the extracted phrases or sentences were identified, and the constructed meanings were described in the form of abstract statements. Categories were formulated based on themes common to all participants, and identified phenomena were described. Afterwards, the findings were reviewed until a consensus was reached among researchers, and two participants validated the findings to confirm that the results were consistent with what they expressed. Lastly, the essence of the phenomenon was described based on agreement among all three authors obtained after a final discussion.

### 2.3. Ethical Considerations

The participants provided written consent after being briefed on the purpose of the study, methods, rights as subjects, and confidentiality when they participated in the primary study. They were made aware of the recording and transcription of interviews, and understood that they had the right to withdraw at any time. Before data collection, institutional review board approval was obtained (No. HYUH 2016-04-045). Obtaining permission from all participants for secondary analysis was difficult as some had stopped visiting the hospital due to a worsening of conditions or death. Hence, the research protocol including secondary use of interview data and waiver of consent was approved by the institutional review board of corresponding author from an affiliated university (No. SWCN-201906-HR-004).

## 3. Results

Among the 10 research participants, 6 were male, 5 had a religion, and 5 were in a spousal relationship (Table 1). The three main themes derived from analysis were: “Frustration with seeing a patient suffering”, “burnout at the cost of a life of dedication”, and “desperate need for help” (Table 2). Details are provided below.

### 3.1. Frustration with Seeing a Patient Suffering

Participants feel sorry to see patients despair and struggle with their condition, and it is painful to think of patients’ hopelessness.

#### 3.1.1. Sadness Emphasizing Patient’s Hopelessness

Patients feel despair after being diagnosed with ALS, and are devastated from not being able to accept reality. Family caregivers are saddened by identifying with the patient’s hopelessness. It pains them to think of the patients giving up hope when they become aware of their worsening condition.

- *“He refused to go to the hospital, and stopped eating. This hurts most. He said he would die anyway, and doesn’t want that (tracheotomy) even if he has trouble breathing.” (Participant 8)*
- *“I don’t want her to see others whose disease had progressed at the hospital. It would only fill her with worry and despair. Just imagine how hopeless she would feel.” (Participant 1)*

#### 3.1.2. Pity Regarding a Patient’s Physical Condition

Family caregivers feel bad over worsening physical symptoms, such as slurred speech and limited movement. The patients interact less with people, and become increasingly isolated. The caregivers expressed compassion for seeing a patient’s deteriorating body and limited social interaction.

- *“He is becoming noticeably thin, being reduced to just skin and bone. When I suggest going out, he doesn’t feel like it because of his miserable state.” (Participant 6)*
- *“He must be feeling frustrated. Not being able to move, that’s the worst part. He had lots of friends. They all left him once he got sick.” (Participant 8)*

### 3.2. Burnout at the Cost of a Life of Dedication

The participants give up their lives to take care of patients, feel sorry for not taking care of other family members, and fear that the patient’s condition may deteriorate and require high-level care. As they continue to take care of the patients, they become exhausted.

#### 3.2.1. Neglecting One’s Own Life

Family caregivers cannot afford to participate in social activities or leisure, and even neglect their own health. Their lives revolve around caregiving, and they leave no time for themselves. They also take over economic activities or household chores that used to be performed by the patient.

- *“I can’t do anything else. I’ve lost my friends. It’s like living in an enclosed space. I don’t have much of a life anymore.” (Participant 8)*
- *“There are household chores to do, as well as my own work. It feels unfair sometimes. It’s all about the patient and children, but never me.” (Participant 10)*

#### 3.2.2. Feeling Sorry for the Family

Family caregivers experience financial difficulties when patients lose their jobs or when they have to give up working to provide caregiving. Family events and child-rearing may be given less priority. This makes the caregivers feel sorry for the entire family.

- *“I should visit my kid’s school, and attend college admissions briefings. I feel sorry for the kids because I don’t have time for all that.” (Participant 2)*
- *“I’m left with so little time to work, so the financial strain is the most difficult.” (Participant 3)*

#### 3.2.3. Worry about Keep Carrying Out the Caregiver Role

Family caregivers are distressed to think of the expected role that they will take on as the patient’s condition worsens. They are worried and frightened about handling medical equipment in the future, for example gastronomy tubes or ventilators.

- *“I’m most worried about how and when he gets the tracheotomy. Yeah, that’s what worries me the most.” (Participant 4)*
- *“I’m worried she’ll be bedridden, that she’ll be hooked up to the ventilator for life. I’m really worried.” (Participant 7)*

#### 3.2.4. Exhaustion from Caregiving

Family caregivers spend the entire day at the side of patients, and cannot afford to fall into deep sleep even at night. They face conflict with patients, who are unable to express themselves due to dysarthria. This makes them feel sorry about the situation, and they regret not committing fully to caregiving.

- *“Because she can’t move. Because I have to spend the entire day helping her up and then back down. She can’t speak as she used to. It gets more difficult over time.” (Participant 7)*
- *“The poor communication with my father worsens the conflict.” (Participant 5)*
- *“I’m stuck in a cycle of giving and giving. It’s no wonder to feel irritated sometimes. I can’t help it when emotions get the better of me.” (Participant 10)*

### 3.3. Desperate Need for Help

The participants are willing to resign themselves to accept the patient’s condition, hoping that the patient will live even if there is no cure, and desperately looking for social support for coping and help for symptom management methods.

#### 3.3.1. Trying to Accept the Situation

Family caregivers try to think that patients were destined for ALS. They are thankful when symptoms do not worsen that much, and adopt an optimistic attitude. They are willing to do their best for patients.

- *“Religion is very comforting. I go to church once a week to pray. I express my gratitude for the past week, and ask for another good week.” (Participant 4)*
- *“It’s my karma and fate. It’s also her karma and fate. If I don’t accept it, it would only make me miserable. I try not to make a fuss, and act like I always do.” (Participant 9)*

#### 3.3.2. Wish to Prolong a Patient’s Survival

Family caregivers try their best to make patients feel comfortable and to lift their mood. Even if there is no cure, they hope for patients to live as long as possible without further deterioration.

- *“We try to attend support group meetings. After all, patients can understand each other best, and find comfort in knowing that there are others going through the same challenges.” (Participant 10)*
- *“I hope it doesn’t get worse. If it stops progressing, maybe it’ll be enough time for a cure to be developed. I just keep hoping.” (Participant 1)*

#### 3.3.3. Seeking Social Support

In spite of the government’s financial support for caregiving, the purchase of medical devices, and hospital expenses, family caregivers are burdened by high medical costs. They expect more social support for caregiving.

- *“The medical costs are expensive. We hired a professional caregiver, and it’s not subsidized by insurance.” (Participant 2)*
- *“We need an electric adjustable bed and other things. They all cost money. A rental service for a hospital bed is not available for Lou Gehrig’s disease.” (Participant 3)*

#### 3.3.4. Finding Better Symptom Management

Family caregivers explore various methods to alleviate symptoms. They try to resolve communication problems due to dysarthria, but cannot obtain a proper solution. They also collect information regarding advanced care, such as preparing for emergency situations.

- *“When I searched on the internet, it showed that protein is good for a patient with Lou Gehrig’s disease. I keep his protein levels up with black beans, soymilk, banana, and walnuts.” (Participant 9)*
- *“I’d like something that helps with communication. For example, a device to write more conveniently, like an iPad, but just could not find a perfect device.” (Participant 5)*

## 4. Discussion

This study conducted descriptive phenomenological analysis of suffering experienced by ALS family caregivers in Korea, and broadly categorized them into three themes: (1) frustration with seeing a patient suffering, (2) burnout at the cost of a life of dedication, and (3) a desperate need for help. The findings of this study provide a broad overview of suffering in relation to ALS family caregivers in Korea. While a previous study on the Korean ALS caregiver experience focused on the perspective of a wife [18], this study expanded to an overview of family distress by analyzing the experiences of various caregivers. While burden and needs were the main themes of a negative experience in a study by Weisser and colleagues [19], interestingly, frustration with seeing a patient suffering was another salient main theme for most of the participants in this study.

Currently, ALS is a disease without a cure [20], thus family members often feel helplessness at not being able to overcome the disease [1,21] and fear impending death upon diagnosis [22]. Furthermore, most participants mentioned they feel pity for the patient as their physical condition worsens, which is similar to witnessing suffering as noted in a study by Galvin et al. [1]. Meanwhile, a majority of participants in this study also expressed empathy for patients. A recent systematic review on the experiences of informal caregivers for patients with ALS reported that they experienced distress and negative emotions, such as anxiety, anger, sorrow, and despair [23], and this study additionally found that family members empathized with the patients. The Korean tradition of familism and strong family bonding [24] are believed to have led to the theme of “sadness emphasizing patient’s hopelessness”. In recent studies of dementia family caregivers, empathy has been associated with a decrease in caregiving burnout, decrease in caregiver distress, and encouragement of caregivers to have more positive caregiving experiences, such as satisfaction with caregiving and positive relationships with people with dementia [25,26]. However, the affective empathy of dementia family caregivers was also found to be related to their high anxiety [27]. Therefore, further research is needed on how ALS family caregivers’ empathy influences their mental health.

As the disease progresses, the primary caregivers’ life revolves around caregiving, typically leaving their own life behind, and the patient becomes the center of their life. This is consistent with the “shift in focus of life” [28] observed in a metasynthesis study of the families of dementia patients in Korea. With the social isolation of ALS patients, caregivers have to be by their side 24/7, and they too become excluded from interactions with society. Such social limitations and restrictions have been established in many previous studies on experiences of ALS caregiving [1,21,29]. Patients become fully dependent on caregivers, and the familial relationship (e.g., husband and wife, mother and daughter) transitions into a patient–nurse relationship [18]. Emotional conflict also arises due to poor communication with patients who have developed speech problems, and repeatedly there are feelings of guilt over disagreements. This is consistent with the findings of factor analysis of a Zarit Burden Interview on ALS caregivers in Korea, which reported “social restrictions”, “anger and frustration”, and “self-criticism” as dimensions of caregiver burden [8]. Furthermore, caregivers also feel guilty for not taking care of teenage children as they should be, and the financial strain results in suffering for the entire family, with caregivers often placing family above their own needs. Most participants also worried about their expected responsibilities in almost professional roles such as handling medical equipment, which suggests improvements are needed to provide additional information, support and compassion for caregivers. With the average age of diagnosis being in the late 50s, patients have to quit their jobs, and their partners have limited time for work due to caregiving. Moreover, the high burden of out-of-pocket money in Korea further exacerbates financial difficulties [9,30].

Korean family caregivers of ALS patients had a strong tendency to accept the disease as fate despite the tough circumstances. Unlike the West, Taoist and Buddhist roots in Korea allow patients and their family to see disease as part of life [31], and this is established as the wisdom of optimistic acceptance of severe illnesses [32]. ALS caregivers in Western countries adopted “living in the moment” as a coping strategy [19,21], and the participants in this study went a step further by accepting the disease as part of their lives. Meanwhile, family caregivers highlighted the need for greater social support, which they found to be lacking despite their efforts to best make use of the available resources. Korean welfare services are standardized according to their classification of physical functioning, not reflecting characteristics of the disease. Patients with ALS experience weakening of the muscles in the arms and legs, but there are also other symptoms not accounted for by the disability classification system. In addition, the current policies do not take into account the rapid rate of progression. For example, patients on crutches may need a wheelchair within the next few weeks. One solution is to improve medical device rental services through ALS associations or community support groups. Since patients require 24-hour observation, a comprehensive community care system should support caregiving over longer hours, so as to ensure sufficient rest time for family and breaks from social isolation.

There are some limitations to our study. First, as we are exploring new questions using secondary data, it is not clear whether data saturation was achieved. Although this is a limitation in the methodology, the use of secondary data also has various strengths. For example, it could relieve the burden of participation from research participants who are “suffering”, and the voice of research participants can be maximized in research and reflected effectively in health policies. Second, this study focused on the meaning of a suffering experience, which limits a wider perspective about the care experience, such as various ranges in terms of special needs. Therefore, further studies are necessary in order to provide more specific measures to meet unmet needs, and thus, lower suffering. It is also necessary to develop scales that measure suffering as well as intervention programs to lessen factors that result in suffering. In such studies, frustration from a patient suffering should be included as an important component, because the caregivers of ALS patients in Korea feel deep empathy and sympathy for patients, which was presented through their sentiments. Health professionals also need to understand what they are suffering from, and should empathize with their difficulties and provide information and resources for better care. In addition, a previous study [33] reported different caregiver burden according to relationship to the patient and demographic characteristics. Future studies should examine family caregivers’ experiences in subgroups (e.g., based on demographics and relationship to the patient).

## 5. Conclusions

Despite the above limitations, the significance of this study lies in its comprehensive description of the meaning of suffering, including the notion of caregiver burden as dealt with in previous studies. Especially, distress from seeing the suffering of a loved one was a particular feature evident in Korean ALS caregivers, reflecting strong family ties. Such high bonding among patients and caregivers should be utilized to enhance care outcomes, and at the same time it is necessary to shift some excessive compassion for patients from caregivers to their own life. For example, the government should provide support to increase the amount of time available to use formal caregivers. Meanwhile, patients are in desperate need of help and support from their families. Thus, it is essential to provide care to lessen the causes of distress and meet the needs of not only patients, but also caregivers through family-centered care. It is also necessary to provide some relief from very challenging life circumstances through measures such as a psychosocial support program.

## Figures and Tables

**Table 1:** Characteristics of participants.

No.	Age (Years)	Sex	Educational Level	Religion	Relation to Patient	Disease Duration (Months)	Disease Type
1	57	F	Middle school	Buddhism	Mother	64	Spinal
2	49	M	College	None	Husband	70	Spinal
3	46	M	High school	None	Husband	71	Bulbar
4	45	M	College	Christian	Wife	62	Bulbar
5	30	M	College	None	Son	23	Bulbar
6	63	F	College	Catholic	Sister	80	Spinal
7	45	M	College	None	Brother	77	Spinal
8	75	F	Elementary school	Buddhism	Mother	78	Spinal
9	58	M	Middle school	Buddhism	Husband	19	Spinal
10	40	F	College	None	Wife	98	Spinal

**Table 2:** Formulated meanings and themes.

Themes	Formulated Meanings
Frustration with seeing a patient suffering	Sadness emphasizing patient’s hopelessness Pity regarding a patient’s physical condition
Burnout at the cost of a life of dedication	Neglecting one’s own life Feeling sorry for the family Worry about keep carrying out the caregiver role Exhaustion from caregiving
Desperate need for help	Trying to accept the situation Wish to prolong a patient’s survival Seeking social support Finding better symptom management

## Data Availability

The data presented in this study are available on request from the corresponding author.
